# Planning ability improves in a yogic education system compared to a modern

**DOI:** 10.4103/0973-6131.41033

**Published:** 2008

**Authors:** R Rangan, H R Nagendra, G Ramachandra Bhat

**Affiliations:** Department of Yoga Research, Swami Vivekananda Yoga Anusandhana Samsthana, Bangalore, India

**Keywords:** Gurukula education system, planning ability, Tower of London test, Vedic chanting, Yoga

## Abstract

**Background:**

Planning skills play a key role in higher developmental processes. The Tower of London test not only measures planning skills, but also the ability to execute plans. Yoga practices aim to bring about higher development. Can a Yoga-based education system be shown to meet this challenge?

**Aim:**

This study was aimed at comparing a Modern Education System (MES) with the ancient Yoga-based system of education, the Gurukula Education System (GES), in developing planning skills.

**Materials and Methods:**

Forty-nine boys with ages ranging from 11 to 13 years were selected from each of two residential schools, one MES and the other GES, providing similar ambience and daily routines. The boys were matched for age and socio-economic status. The GES educational program is based around integrated yoga modules while the MES provides a conventional modern education program. Planning and executive abilities were assessed using the Tower of London test at the start and the end of an academic year.

**Results:**

Within groups, the pre-post test differences were significant for both groups. However, the between-groups results showed improvement in the GES group compared to the MES group at a *P* < 0.001 significance level.

**Conclusions:**

The study suggests that whereas both MES and GES Yoga-based education improve planning and execution skills in school boys, GES is more effective of the two systems.

## INTRODUCTION

It is important for India to enhance national levels of achievement in education by improving its system of modern education, including increasing levels of academic achievement and decreasing levels of stress and hypertension among students. This paper is the first in a series of four, which examine the traditional Gurukula Education System (GES) for ways in which its practices might contribute advantageously to the Modern Education System (MES). The concern of this first paper is with the development of planning abilities associated with self-reliant, successful persons.

The GES is a Vedic system of learning, which includes many yogic practices. It is time-tested and has been preserved for several millennia in an unbroken tradition. In the Indian tradition, it is believed that GES brings great benefits to society, including improvements in cognitive and higher mental abilities.[1] Although the GES stands in the heart of tradition, it is yet to be scientifically examined to understand its advantages.

Outside the context of education, studies have been conducted on many different yogic practices. These include asanas, pranayama, Vedic chanting, and meditation, and reveal that such techniques can be used as intervention to enhance cognitive abilities such as attention, concentration,[2] and visual and spatial memory.[3] A study of planning ability conducted by Manjunath and Telles in students in a girls' school used the Tower of London test before and after an intervention of two months' practice of yoga for 75 minutes per day. The students who practised yoga gave significantly better results than those who performed physical exercises.[4]

Kadambini used the same Tower of London test on 30 school students in an RCT (Randomised Control Trial) design to assess the effects of a short-term intervention of a nine day period practising yoga lifestyle including intensive yoga practice.[5] The yoga group showed significantly greater improvement in their planning ability in contrast to controls. Both these studies indicate that Yoga interventions can be effective in improving planning ability.

Planning has been defined as identification and organization of the steps and elements needed to carry out an intention or achieve a goal.[6] It is a complex function with many components such as speed of processing, mental flexibility, working memory, regulation of thoughts, and error correction.[7] Planning ability depends mainly on the functioning of the prefrontal cortex.[8]

The Tower of London test is a standardized test for measuring planning ability.[9] The test evaluates the subjects' ability to plan and anticipate results of their actions to achieve a predetermined goal. The present study extends the work of Manjunath and Kadambini by assessing the effect of a *one year* Yoga intervention on boys in a GES school compared to a matched control group of boys in a MES school.

## MATERIALS AND METHODS

### Subjects

Two residential schools, one MES and the other GES providing similar ambience and daily routines, were chosen. Both the residential schools had similar natural surroundings with an atmosphere congenial for learning. Out of the 110 students in the Yoga-based boys' Gurukula (GES) and 500 students studying in the MES, a group of 49 healthy boys between 11 and 13 years of age from each school, were one-to-one matched for age, family circumstances and socio-economic background.

The boys' health status was assessed by a doctor based on their personal history and a general clinical examination. Those having congenital defects or taking medication known to affect planning or cognitive abilities were excluded from the study. The students in the GES school were all freshers and had received a modern education similar to the MES school until that time, when being interested in GES, they had chosen the Gurukula school. An independent samples‘t’-test on the baseline data showed no significant differences (P > 0.05) between the two groups for any of the demographic parameters. The demographic data for the two groups are presented in Table 1.

**Table 1:** Demographic data of boys studying in MES and GES schools

	Details of students	RS	Years	Years				
		S	A	B	C	D	E	Age
Groups	*n*	(mean±sd)	(mean±sd)	(mean±sd)	(mean±sd)	(mean±sd)	(mean±sd)	(mean±sd)
		6448.98 ±	1.31 ±	1.18 ±	2.18 ±	4.02 ±	1.35 ±	12.16 ±
GES	49	1969.15	1.37	0.39	1.52	0.14	0.48	0.66
		6704.08 ±	0.47 ±	1.18 ±	2.35 ±	4.02 ±	1.33 ±	12.31 ±
MES	49	2174.47	0.53	0.39	1.38	0.14	0.47	0.68

GES: Gurukula education system, MES: Modern education system

A-Education of father, B-Education of mother, (Education up to SSLC–1, Graduation–2, Post graduation–3, Professionals–4) C: Occupation of father, D: Occupation of mother (Agriculture-1, Business-2, Academician-3, Others-4) E: social setup (Rural-1 Urban-2)

The results show no significant differences between GES and MES in all the demographic parameters (Independent samples † test P > 0.05). Differences between the GES and MES groups for levels of education of father and mother, occupation of father and mother and social setup were assessed using X^2^ test and were found to be not significant (*P* > 0.05).

### Assessments

Shallice's Tower of London test was used to assess planning abilities and execution skills.[9] The Tower of London test requires the subject to move an array of colored discs mounted on three vertical rods, to match a particular goal arrangement given in a picture. Each subject has to complete four tasks at increasing levels of complexity; the first level requires two moves to reach the goal, the second three moves, the next four and the fourth level, five moves. Subjects are assessed on planning time, execution time, mean total time, and the number of moves. Both groups were assessed before and after the academic year. All assessments were made before lunch. Time and day of assessments were the same for matched pairs. Test instructions were given in English in both schools.

### Significance of planning time

The measure of planning time assesses planning skill. The essence of planning is to see how to attain a goal through a series of intermediate steps. The subject plans in advance the complete sequence of moves required to solve the problem. In order to do so, the subject considers the consequences of various courses of action.[10] Goal setting involves not only identifying the final goal, but also any necessary intermediate goals. Thus, assessment of planning time evaluates both these faculties: setting the goal and intermediate steps.

### Significance of execution time

Measuring execution time not only assesses motor skill but also the planning ability. This is because new decisions can occur during execution. Planning ability not only sets goals, but also monitors performance to reach a goal and make corrections to the chosen course, in order to ensure that the goal is attained.[10] In carrying out a planned strategy, frontal association areas of the brain cortex are used to execute complex functions such as delayed response motor tasks and changing strategies, if and when necessary.[11] Thus, assessing execution time can evaluate motor skills and planning ability related to frontal association areas.

### Significance of number of moves

Assessing the number of moves evaluates perfection of planning. If planning has not been perfect, the number of moves increases. This is also a measure of brain function. Imaging studies have found that more efficient planning involving fewer moves is associated with increased activation of the left prefrontal cortex.

### Masking

One-to-one matching of students was done under the guidance of a statistician. Demographic data were collected by trained persons not involved in the design of the study. The Tower of London test assessments were carried out under the guidance of a psychologist, by trained persons who had not been involved in the selection process, and who did not know the design of the study. No teacher at either school was involved in making the assessments. There were no interactions between the GES and MES schools as they were in different locations more than 100 kilometers apart. Furthermore, no one at either school knew the identity of the other school.

### Intervention

The GES School used an educational program which integrated yoga practices, while the MES provided a conventional modern education program. The GES program included yogic postures (asanas), voluntary regulated breathing (pranayama), meditation (dhyana), recitation of mantras (japa), yogic prayers, worship (puja) and Yogic games (team games played on Yogic principles). In contrast, the MES program included physical exercises, mathematical puzzles, music, prayer and normal sports. The daily routines of the two schools are shown in Table 2.

**Table 2:** Daily routine in the two residential schools

GES		MES	
	
Time	Schedule	Time	Schedule
5:00 AM	Wake up	5:00 AM	Wake up
5:30–5:45 AM	Meditation and Pranayama	5:00–5:50 AM	Ablutions
5:45–6:15 AM	Yogasanas	6:00–6:15 AM	Prayer
6:15–6:45 AM	Cleaning	6:15–6:45 AM	Physical exercises
6:45–7:30 AM	Ablutions	7:00–7:55 AM	Self-study
7:40–8:00 AM	Puja		
8:30–9:15 AM	Breakfast	8:00–9:15 AM	Breakfast/cleaning
9:30–10:30 AM	Vedic chanting	9:30 AM–12.00 PM	Sessions
10:30 AM–12:45 PM	Sessions	12.00–1:00 PM	Music
1:00–2:30 PM	Lunch	1:00–2:20 PM	Lunch/rest
2:45–4:45 PM	Sessions	2:30–4:30 PM	Sessions
4:45–5:00 PM	Snacks	4:45–5:00 PM	Snacks
5:00–6:00 PM	Tuning to nature	5:00–6:00 PM	Tuning to nature
6:00–6:45 PM	Yogic games	6:15–6:45 PM	Games
6:45–7:00 PM	Meditation and pranayama	6:45–7:00 PM	Prayer
7:00–8:00 PM	Self-study	7.00–8.30 PM	Self-study
8:00–9.00 PM	Dinner	8:30–9:15 PM	Dinner
9:00–10.00 PM	Self-work	9:15–10:00 PM	Self-work
10.00 PM	Lights off	10:00 PM	Lights off

GES: Gurukula Education System, MES: Modern education system.

### Data analysis

The pre- data of the two groups were compared using an Independent Samples ‘t’ test. The Kolmogorov test of normality showed that the pre- data were not normally distributed, as would be expected, because the Tower of London test has distinct minima but no upper limits. Hence, nonparametric tests were used in the analysis. Within groups, the pre-post data were analyzed using the Wilcoxon Signed Ranks Test, while between groups the pre-post data were analyzed using the Mann-Whitney U Test. SPSS 10.0 was used for analysis.

## RESULTS

The results of tests are summarized in Table 3. Both groups of students performed similarly on the pre- test at the start of the academic year (pre- data), an Independent Sample's ‘t’ Test found no significant difference between the two groups. This also means that it was legitimate to subsequently compare their performances. The Wilcoxon Signed Ranks test comparing the pre-post values within the groups showed that improvements in both groups were significant at P < 0.005.

**Table 3:** Tower of London test contrasting GES with MES schools

*n* = 49		GES			MES		
			
	Trials	Pre (mean ± SD)	Post (mean ± SD)	% changes	Pre (mean ± SD)	Post (mean ± SD)	% changes	Between groups significance
Planning time (secs)	2	4.35±0.63	3.73±0.57*	14.253	4.31±0.55	3.94±0.52**	8.584	0.061
	3	6.45±0.84	6.08±0.40*	5.736	6.57±0.50	6.18±0.53*	5.936	0.250
	4	9.59±0.57	9.22±0.55*	3.858	9.80±0.58	9.47±0.62*	3.367	0.034**
	5	12.73±0.070	12.39±0.64*	2.670	12.78±0.77	12.53±0.71*	1.956	0.196
Execution time (secs)	2	4.90±0.47	4.22±0.42*	13.877	4.92±0.45	4.43±0.58*	9.959	0.030**
	3	7.43±0.61	6.69±0.58*	9.959	7.22±0.59	6.90±0.42*	4.432	0.036**
	4	11.08±0.86	10.24±0.69*	7.581	11.04±0.82	10.51±0.65*	4.800	0.025**
	5	15.51±1.49	14.98±0.80*	3.417	15.63±0.88	15.10±1.14*	3.390	0.125
Mean total time (secs)	2	9.24±0.85	7.95±0.76	13.961	9.22±0.71	8.37± 0.83*	9.219	0.011**
	3	13.88±0.78	12.78±0.79*	7.925	13.80±0.69	13.08±0.73*	5.217	0.040**
	4	20.67±1.16	19.47±0.96*	5.806	20.84±1.12	19.98±0.90*	4.126	0.004**
	5	28.24±1.35	27.37±1.24*	3.080	28.41±1.29	27.63±1.47*	2.745	0.103
Number of moves	2	2.39 ± 0.36	2.11 ± 0.26*	11.715	2.28±0.31	2.22 ± 0.31	2.631	0.051**
	3	3.35 ± 0.40	3.09 ± 0.35*	7.761	3.31±0.37	3.12±0.21*	5.740	0.084
	4	4.26 ± 0.41	4.16 ± 0.30	2.347	4.37±0.46	4.20±0.37*	3.890	0.661
	5	5.32 ± 0.37	5.07±0.14*	4.699	5.25±0.32	5.06±0.30*	3.619	0.880

* *P* < 0.05, Wilcoxon signed ranks test, comparing pre- and post- values within groups

** P < 0.05, Mann Whitney U test comparing the two groups (Mean time = Planning time + execution time, Number of moves = Number of moves to complete the task) (GES: Yoga-based (Gurukula) system, MES: Modern education system)

*P < 0.05, Wilcoxen signed ranks test and **P <0.05, Mann-Whitney U test

(GES was significantly better than MES in the trial four of the planning time, trial two and trial three and trial four of execution time and in the trial two in mean moves.) (*P* < 0.005, Mann-Whitney U test)

The Mann-Whitney U Test used to compare results between the two groups, showed significant differences on three of the four subscales of planning, execution, and mean time, and only one of the subscales of number of moves. In all cases, it was the smaller number of moves that were significant. Between-groups results showed that the greater improvement in GES compared to the MES group was significant in trial four of the planning time, trials two, three and four of execution time, and in trial two in the number of moves (*P* < 0.005, Mann-Whitney U test).

## DISCUSSION

The most prominent result is that both systems of education improved planning and execution abilities. How much of this improvement could be attributed to the natural increase in IQ in early teenagers is not clear. In most of the Tower of London test subscales, both level of performance and % improvement were greater for the GES group compared to the MES group: 15 out of 16 subscales for level of performance, and 14 out of 16 subscales for % improvement. However, the four mean total time subscales are not statistically independent. Applying the nonparametric sign test to the data for the remaining twelve subscales yields a significance of *P* < 0.003 for better performance, and *P* < 0.016 for greater improvement for GES students compared to MES students. At the end of the year, the GES students had definitely improved more and performed better on the test.

The only significant comparative result in Planning Time was for the 4-move trial. In contrast, for Execution Time, significant results were achieved for the 2, 3, and 4 move trials: while both groups took much the same time to plan, the GES group executed each task more quickly. Overall, the two most significant differences were *P* < 0.004 for trial 4 of mean total time, and *P* < 0.011 for trial 2 of the same subscale. The results of the GES group's yoga practice translated into increased accuracy of planning, improved speed of action, and to some extent, more precise task execution.

Although results reached significance in these cases, no significance was found between the groups for the number-of-moves assessment, but the *n* = 2 result was of borderline significance, and three out of the four showed higher values for GES compared to MES.

Similarly the five-moves' task showed no significant differences between the two groups, though the GES group consistently improved more than the MES group, which in itself, was a result of borderline significance. There may be various reasons for this. In harder problems, more complex planning is required and creates difficulties.[9] All the subjects found the five-moves' task complicated and only a few completed the task in five moves.

These results beg the question as to why the practices in the GES school should have produced the differences observed. The GES yoga-based lifestyle includes most key features of Yoga: Asanas, physical postures, which produce relaxation at a bodily level; Pranayama; voluntary regulation of breathing, which is designed to calm down the breath; Dhyana, generally understood in this context as an internal Japa or repetition of a Mantra which calms the mind; Yogic prayers and Puja, or worship, which culture and balance the emotions; Yogic games, a set of games which not only stimulates but also relaxes and expands the mind to a state of greater freedom. The overall purpose of incorporating these Yogic features into the students' lives is to learn to perform all actions against a back-drop of stress-free states of the mind. The intention is that such stress-free patterns of functioning should carry over into other activities such as academic tests, examinations and professional life in later years. These activities and purposes are characteristically different from the nonyogic MES school. Based on these fundamental principles, different results might be expected.

The first observation is that the Tower of London test depends on mental speed in both planning and execution. Which of the above practices in GES might develop mental alertness and speed? Planning is a central multi-component process mediated by the prefrontal cortex, and is involved in the execution of nonroutine actions. Several of the practices lead practitioners to peace and tranquillity of the mind, which might seem contrary to speed, but in general, a settled mind has greater clarity of thinking, and can function more quickly. A few GES practices like Kapalabhati (a breathing exercise), Bhajans (harmonious songs of expansion and surrender), and Yogic games have the element of speed and stimulation; but their objective is also mental calmness and peace. This is because, although they start with speed-related stimulation, they end in a state of relaxation and silence. That may be why the comparative results were initially significant, but why as the number of moves increased, they gradually decreased to insignificance at five moves. It would appear that although the GES group could execute their plans more quickly, they were reduced to almost the same speed as the MES group when they had to think about complicated tasks.

This suggests that the GES group were better able to visualize their plan and hold it in mind. Interestingly, such an improved ability to hold an idea in the mind is a well-known result of the practice of Transcendental Meditation (TM). TM has been shown in several studies to strongly improve the property of field independence where a particular property or pattern has to be identified against a confusing background.[12]

With regard to Execution Time, increased alertness may also be expected to improve performance. In particular, in trials with 2, 3 and 4 moves, the GES group perceived wrong moves quickly and were able to make the correct move without much delay. In addition, the task cannot be achieved efficiently without growth of the Supervisory Attentional System (S.A.S.), which contains the general programming or planning systems that operate on schemas in every domain. The data suggest that the S.A.S. is more developed by the GES curriculum.[13]

Earlier studies of the Tower of London test by Manjunath *et al.*[4] and Kadambini[5] compared interventions of yoga practices (Asanas and Pranayama) with physical exercises. Both were randomised control trial studies and found higher levels of planning ability in the yoga group. The present study is consistent with these findings.

It is pertinent to compare these results with those found for various kinds of meditation. Several studies have shown that the practice of the Transcendental Meditation technique produces a state of restful alertness, which enhances cognitive and functional abilities.[14] A positron emission tomography (PET) study of regional cerebral metabolic responses showed that ratios of frontal vs occipital responses were significantly higher during yoga relaxation in eight practitioners.[15] A Functional Magnetic Resonance Imaging (fMRI) study showed that the dorsolateral prefrontal cortex is among the brain regions activated during meditation.[13] This raises the question of which brain regions are involved in performing the Tower of London test.

Although the Tower of London test is now used to study planning ability in normal persons,[16] Shallice originally developed it to investigate planning abilities in patients with frontal lobe damage; this correlated with poor test performance.[9] His lesion studies showed that left frontal lesions are associated with planning deficiencies.[9] Other studies found that inappropriate organization associated with poor planning is increased by bilateral prefrontal lesions.[17] The dorsolateral prefrontal cortex is associated with the components of generating, selecting and / or remembering mental moves.[18]

Single photon emission computerized tomography (SPECT) has shown that the level of regional cerebral blood flow increases in the left prefrontal cortex in normal persons during Tower of London test performance.[16] Improvements in Tower of London task performance seen in the present study following a year of GES, suggest that the Yogic system of GES improves left frontal lobe function more than MES. If this is the case, the question is: which prefrontal cortex functions does it improve?

According to Morris *et al.*, planning during the Tower of London test activates a wide network consisting of the dorsal prefrontal cortex, premotor and parietal cortex, and the cerebellum.[16] The association of the dorsolateral prefrontal cortex with generation, selection, and memory of mental moves,[18] suggests that it may also be involved in improving GES students' performance on the test. Similarly, another study has related to the growth of planning ability and attention to higher fractional anisotropy and lower apparent diffusion coefficient;[19] both these may also be increased in GES students.

While the strength of the study could have been enhanced by an RCT design, the practical limitations of such trials are obvious. Individual component analysis of different practices such as meditation, pranayama or chanting might throw more light on the mechanisms whereby GES generates more benefits than MES.
